# The Prognostic Role of Blood Inflammatory Biomarkers and EGFR Mutation Status in Stage IIIA/N2 Non-Small Cell Lung Cancer Patients Treated With Trimodality Therapy

**DOI:** 10.3389/fonc.2021.707041

**Published:** 2021-11-30

**Authors:** Hui Yang, Kunlun Wang, Bingxu Li, Shenglei Li, Yan Li, Ling Yuan

**Affiliations:** ^1^ Department of Radiation Oncology, The Affiliated Cancer Hospital of Zhengzhou University, Zhengzhou, China; ^2^ Department of Radiation Oncology, Anyang Cancer Hospital, The Forth Affiliated Hospital of Henan University of Science and Technology, Anyang, China

**Keywords:** stage IIIA/N2, non-small cell lung cancer, blood inflammatory biomarkers, systemic immune-inflammation index, systemic inflammation response index

## Abstract

**Objectives:**

Various blood inflammatory biomarkers were associated with treatment response and prognosis of non-small cell lung cancer (NSCLC) in previous studies. In this study, we retrospectively evaluated the prognostic role of pretreatment blood inflammatory biomarkers and epidermal growth factor receptor (EGFR) mutation status in stage IIIA/N2 NSCLC patients with trimodality therapy.

**Methods:**

Completely resected stage IIIA/N2 NSCLC patients with adjuvant chemotherapy and postoperative radiotherapy (PORT) were assessed in this study. Cutoff values of blood inflammatory factors were calculated by the R package SurvivalROC of R software. SPSS Statistics software was used for survival analyses. Kaplan-Meier survival curve and log-rank test were used to compare the survival difference between every two groups. Univariate and multivariate analyses of predictive factors were performed by Cox proportional hazards regression model.

**Results:**

The univariate analysis showed that T stage (p=0.007), EGFR mutation status (p=0.043), lymphocyte-to-monocyte ratio (LMR) (p=0.067), and systemic immune-inflammation index (SII) (p=0.043) were significant prognostic factors of disease-free survival (DFS). In the multivariate analysis, T2 (HR=0. 885, 95% CI: 0.059-0.583, p=0.004), EGFR mutation-positive (HR=0.108, 95% CI: 0.023-0.498, p=0.004) and elevated pretreatment SII (HR=0.181, 95%CI: 0.046-0.709, p=0.014) were independently related to shorter DFS. High pretreatment neutrophil counts (HR=0.113, p=0.019) and high systemic inflammation response index (SIRI) (HR=0.123, p=0.025) were correlated with worse overall survival (OS) by the univariate analysis. In the multivariate analysis, only high pretreatment SIRI was an independent predictor for poorer OS (HR=0.025, 95% CI: 0.001-0.467, p=0.014).

**Conclusions:**

In conclusion, we identified that high pretreatment SII and SIRI were unfavorable prognostic factors in stage IIIA/N2 NSCLC patients treated with surgery, adjuvant chemotherapy and PORT. Patients with high pretreatment SII, high pretreatment SIRI, T2, and EGFR mutation-positive may need more forceful adjuvant treatment. Further prospective studies with large-scale are needed to validate our results and identify the proper cut-off values and optimum adjuvant treatment for distinct patient population.

## Introduction

Among the most common cancers, lung cancer ranks first in cancer-associated death worldwide (1). More than 80% of lung cancer patients are non-small cell lung cancer (NSCLC), and 15% of them were diagnosed as stage IIIA (2). Furthermore, stage IIIA NSCLC patients have heterogeneous clinical features and prognoses. Stage IIIA/N2 NSCLC patients were always the hotspot of study and require multidisciplinary treatment approaches. For unresectable patients, definitive chemora-diotherapy (CRT) followed by maintenance durvalumab was preferred according to the PACIFIC Trial (3). For resectable disease, surgery followed by adjuvant chemotherapy with or without postoperative radiotherapy (PORT) was recommended by the National Comprehensive cancer network (NCCN).

However, at least 30% of stage IIIA/N2 NSCLC patients suffered recurrence or metastasis within five years after complete surgical resection (4). Plenty of randomized controlled trials (RCT) and large-scale retrospective studies revealed the importance of PORT in stage IIIA/N2 NSCLC after complete surgical resection (5–10). PORT not only improved disease-free survival (DFS) but also increased the overall survival time (OS) of patients. The NCT00880971 trial founded that the PORT group had a better 3-years local recurrence-free rate (69.8 *vs* 62.4%, p=0.03) than chemotherapy alone after complete surgery (11). However, the median DFS (26.5 vs 22.7 months, p=0.10), and median OS (not reached vs 90.9 months, p=0.94) had no significant difference between the two groups. The Lung ART study (NCT00410683) also showed that PORT reduced the local recurrence rate (from 46.1 to 25.0%), but could not bring DFS or OS benefit (12). The adjuvant treatments for stage IIIA/N2 patients are still controversial now. Trimodality therapy (surgery, adjuvant chemotherapy and PORT) is one of the suitable treatment modalities so far.

Inflammation response in the tumor environment was closely related to tumor development, growth, and recurrence (13, 14). The peripheral blood cells, including neutrophils, lymphocytes, monocytes, and platelets, play an essential role in the inflammatory response and reflect the anti-tumor immunity in the host (15–17). Based on previous studies, the count of these cells and their ratios are correlated with treatment response and prognosis of cancer patients. Hence, neutrophil counts, lymphocyte counts, monocyte counts, platelet count, neutrophil-to-lymphocyte ratio (NLR), platelet-to-lymphocyte ratio (PLR), lymphocyte-to-monocyte ratio (LMR), systemic immune-inflammation index (SII), and systemic inflammation response index (SIRI) have been identified as blood inflammatory biomarkers and served as prognostic indicators in various cancers (18–22).

Concerning lung cancer, the prognostic role of blood inflammatory biomarkers has been described in previous studies. A meta-analysis reported that higher pretreatment NLR was a significant predicator of poor survival not only in patients treated with chemotherapy but also in patients with immunotherapy or targeted therapy (23). High pretreatment NLR, high PLR, and low LMR were also related to poor outcomes in early stage, locally advanced and advanced NSCLC patients with diverse treatments (24–27). Another study of stage III NSCLC patients treated with concurrent CRT found that high pretreatment SII was independently correlated with chemoradiation resistance and poor prognosis (28). SIRI was also important in unresectable stage III NSCLC patients treated with CRT (29) and advanced NSCLC patients treated with epidermal growth factor receptor (EGFR) tyrosine kinase inhibitors (TKIs) (30).

Since blood inflammatory biomarkers were associated with the prognosis of patients, these factors were crucial to identify high-risk patients and make optimum treatment decision for them. A retrospective study of locally advanced NSCLC patients treated with preoperative CRT and surgery detected that high postoperative NLR was an indicator for poor prognosis (31). Postoperative adjuvant chemotherapy significantly increased the 5-year OS rate in patients with high postoperative NLR (cutoff value: 4.06, p=0.016), and could not improve the prognosis of patients with low NLR (p=0.19) (31). Hence, patients with high postoperative NLR had a poor prognosis, and need postoperative adjuvant chemotherapy to improve the survival time. For patients with low NLR, preoperative CRT and surgery may be enough.

However, the prognostic impact of blood inflammatory biomarkers in stage IIIA/N2 patients treated with trimodality therapy is still unclear. In this study, we retrospectively evaluated the pretreatment blood inflammatory biomarkers and other clinicopathological factors to find potential prognostic biomarkers and identify the high- risk patients who need more forceful adjuvant treatment.

## Materials and Methods

### Patient Selection

This study retrospectively screened patients diagnosed in the Affiliated Cancer Hospital of Zhengzhou University from January 2015 to December 2019. The inclusion criteria were: stage IIIA/N2 according to the 8th edition of the American Joint Committee on Cancer (AJCC) cancer staging system; completely resected; no neoadjuvant therapy; received four cycles of postoperative chemotherapy and radiotherapy after surgery; no history of other malignant tumors. Patients were excluded when disease progression occurred before the completion of postoperative treatment; postoperative treatment was not completed; or without enough follow-up data.

### Data Collection

We collected medical records from the hospital database, including age, gender, pathological type, tumor size, positive lymph nodes, EGFR mutation status, and radiation sequence with chemotherapy (concurrent, sequential). Pretreatment blood inflammatory biomarkers including neutrophil counts, lymphocyte counts, monocyte counts, and platelet count were assumed from routine laboratory results within one week before surgery. NLR=neutrophil count/lymphocyte count; LMR=lymphocyte count/monocyte count; PLR=platelet count/lymphocyte count; SII=platelet count × neutrophil counts/lymphocyte counts; SIRI=neutrophil count × monocyte count/lymphocyte count.

### Statistical Analysis

DFS was defined as the duration from surgery to disease progression, or death. OS was established as the time from surgery to cancer-associated death or the date of the last follow-up.

R software (version 4.0.4) and SPSS Statistics software (version 26.0) were used for the analysis in this study. Cutoff values of blood inflammatory factors were calculated by the R package SurvivalROC through receiver operating characteristic (ROC) curves. The SurvivalROC package iteratively tests all cutoff values to find the cut-point that achieves the maximum log-rank statistic. Corresponding two-tailed p values were measured, and a p-value of <0.05 was considered statistically significant. Kaplan-Meier survival curve and log-rank test were used to compare the survival difference between the two groups. Univariate and multivariate analyses of predictive factors were performed by Cox proportional hazards regression model. Proper factors with p<0.1 in univariate analysis were selected into multivariate analysis to validate independent prognostic factors. The results of prognostic factors were expressed as hazard ratio (HR) with a 95% confidence interval (CI).

## Results

A total of 34 patients were collected in this study. The median follow-up time was 26.4 months (12.4 to 55.4 months). Median DFS was 38.7 months (95% CI: 28.7-48.7 months) and median OS was 52.7 months (95% CI: 48.3-57.1 months) in all patients. Patients had a median age of 56.5 years (range 38–73 years) at the time of diagnosis. Eleven patients (32.4%) were older than 60 years, while 23 patients (67.6%) were under 60 years. Our patients were predominantly adenocarcinoma (91.2%), only three patients were squamous carcinoma or others. 21 patients were T1 (T1a, 2 patients; T1b, 7 patients; T1c, 12 patients) and 13 patients were T2 (T2a, 11patients; T2b, 2 patients). 41.2% of patients had multistation N2 lymph node, other 20 patients were single-station N2. The EGFR mutation status showed that 61.8% of patients were EGFR mutation-negative. 67.6% of patients underwent sequential chemoradiotherapy, only 11 (32.4%) patients had concurrent chemoradiotherapy (**Table 1**).

**Table 1:** Baseline characteristics of all patients (n=34).

Characteristics	Number (%)
Gender	
Male	20 (58.8)
Female	14 (41.2)
Age (year)	56.5 (38–73)
≤60	23 (67.6)
>60	11 (32.4)
Histological subtype	
Adenocarcinoma	31 (91.2)
Squamous carcinoma and others	3 (8.8)
Pathological stage	
1	21 (61.8)
1a/1b/1c	2 (5.9)/7 (20.6)/12 (35.3)
2	13 (38.2)
2a/2b	11 (32.4)/2 (5.8)
N2 lymph node	
Multistation	14 (41.2)
Single-station	20 (58.8)
EGFR mutation	
Positive	13 (38.2)
Negative	21 (61.8)
Radiation sequence	
Concurrent	11 (32.4)
Sequential	23 (67.6)

### Cutoff Values of Blood Inflammatory Biomarkers

ROC curves were generated to determine the cutoff values of blood inflammatory biomarkers. The cutoff values based on DFS for pretreatment neutrophil counts, lymphocyte counts, monocyte counts, platelet counts, NLR, PLR, SII, and SIRI were 3.97, 1.86, 0.35, 253, 2.34, 134.68, 7.46, 708.40, and 0.77, respectively. The cutoff values based on OS were 5.77, 1.86, 0.25, 363, 2.80, 77.06, 7.44, 918.63, and 0.82, respectively. Patients were divided into two groups based on the corresponding cutoff values.

### Univariate and Multivariate Analysis of DFS

**Figure 1:**
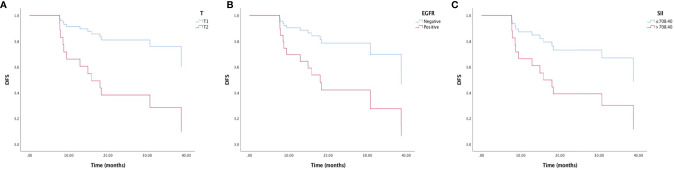
Kaplan-Meier survival curve of disease-free survival (DFS) and **(A)** T stage, **(B)** EGFR mutation status, and **(C)** immune-inflammation index (SII).

The univariate analysis and Kaplan-Meier survival curve showed that T stage (HR=0.220, 95% CI: 0.073-0.663, p=0.007), EGFR mutation status (HR=0.278, 95% CI: 0.081-0.959, p=0.043), LMR (HR=0.358, 95% CI: 0.119-1.075, p=0.067), and SII (HR=0.334, 95%CI: 0.116-0.964, p=0.043) were significantly associated with DFS (**Figure 1**). In the multivariate analysis, T2 (HR=0. 885, 95% CI: 0.059-0.583, p=0.004), EGFR mutation-positive (HR=0.108, 95% CI: 0.023-0.498, p=0.004) and elevated pretreatment SII (HR=0.181, 95%CI: 0.046-0.709, p=0.014) were independent predicators for poor DFS. The role of pretreatment neutrophil counts, lymphocyte counts, monocyte counts, platelet counts, NLR, PLR, and SIRI could not be identified in our study (**Table 2**).

**Table 2:** Univariate and multivariate analyses of DFS.

	Parameter	Univariate	p	Multivariate	p
		HR (95%CI)		HR (95%CI)	
Radiation sequence	Concurrent vs. Sequential	0.630 (0.196-2.019)	0.436		
Gender	Male vs. Female	0.509 (0.176-1.473)	0.213		
Age	≤60 vs. >60	3.168 (0.704-14.258)	0.133		
Histological subtype	Adenocarcinoma vs. Squamous carcinoma	1.876 (0.244-14.436)	0.546		
T stage	T1 vs. T2	0.220 (0.073-0.663)	**0.007**	0.885 (0.059-0.583)	**0.004**
N2 station	Single vs. Multistation	1.072 (0.367-3.134)	0.899		
EGFR mutation	Negative vs. Positive	0.278 (0.081-0.959)	**0.043**	0.108 (0.023-0.498)	**0.004**
Neutrophil counts	≤3.97 vs. >3.97	0.550 (0.187-1.613)	0.276		
Lymphocyte counts	≤1.86 vs. >1.86	0.419 (0.131-1.340)	0.142		
Monocyte counts	≤0.35 vs. >0.35	0.339 (0.107-1.076)	0.066		
Platelet counts	≤253 vs. >253	0.313 (0.096-1.024)	0.055		
NLR	≤2.34 vs. >2.34	0.620 (0.214-1.795)	0.378		
PLR	≤134.68 vs. >134.68	0.662 (0.232-1.894)	0.442		
LMR	≤7.46 vs. >7.46	0.358 (0.119-1.075)	**0.067**		
SII	≤708.40 vs. >708.40	0.334 (0.116-0.964)	**0.043**	0.181 (0.046-0.709)	**0.014**
SIRI	≤0.77 vs. >0.77	0.610 (0.182-2.043)	0.423		

Bold values means p values < 0.05.

### Univariate and Multivariate Analysis of OS

**Figure 2:**
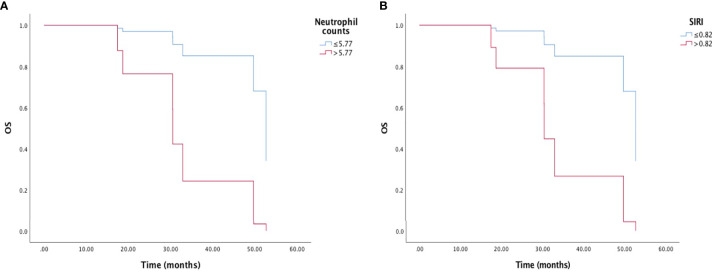
Kaplan-Meier survival curve of overall survival (OS) and **(A)** neutrophil counts, and **(B)** inflammation response index (SIRI).

High pretreatment neutrophil counts (HR=0.113, 95% CI: 0.018-0.699, p=0.019) and high SIRI (HR=0.123, 95% CI: 0.020-0.722, p=0.025) were related to worse OS by the univariate analysis and Kaplan-Meier survival curve (**Figure 2**). In the multivariate analysis, only high pretreatment SIRI was an independent predictor for shorter OS (HR=0.025, 95% CI: 0.001-0.467, p=0.014). EGFR mutation and SII were likely to influence OS, but did not reach statistical significance (**Table 3**).

**Table 3:** Univariate and multivariate analyses of OS.

	Parameter	Univariate	p	Multivariate	p
		HR (95%CI)		HR (95%CI)	
Radiation sequence	Concurrent vs. Sequential	1.768 (0.290-10.762)	0.536		
Gender	Male vs. Female	0.766 (0.170-3.448)	0.729		
Age	≤60 vs. >60	0.845 (0.155-4.607)	0.846		
Histological subtype	Adenocarcinoma vs. Squamous carcinoma	35.226 (0.008-159235.874)	0.407		
T stage	T1 vs. T2	0.959 (0.192-4.793)	0.959		
N2 station	Single vs. Multistation	2.352 (0.454-12.189)	0.308		
EGFR mutation	Negative vs. Positive	0.190 (0.034-1.058)	0.058		
Neutrophil counts	≤5.77 vs. >5.77	0.113 (0.018-0.699)	**0.019**		
Lymphocyte counts	≤1.86 vs. >1.86	0.900 (0.200-4.049)	0.891		
Monocyte counts	≤0.25 vs. >0.25	0.631 (0.117-3.401)	0.591		
Platelet counts	≤363 vs. >363	0.157 (0.016-1.536)	0.112		
NLR	≤2.80 vs. >2.80	0.474 (0.779-28.57)	0.091		
PLR	≤77.06 vs. >77.06	1.088 (0.126-9.434)	0.939		
LMR	≤7.44 vs. >7.44	0.894 (0.194-4.127)	0.886		
SII	≤918.63 vs. >918.63	0.185 (0.031-1.116)	0.066		
SIRI	≤0.82 vs. >0.82	0.123 (0.020-0.772)	**0.025**	0.025 (0.001-0.467)	0.014

Bold values means p values < 0.05.

## Discussion

Stage IIIA/N2 NSCLC patients have a high risk of local progression and distant metastasis after complete surgical resection. After definitive treatment, some tumor cells still exist in the host microenvironment and cause recurrence and metastasis soon. Previous RCTs have revealed the importance of adjuvant treatment in the distinct patient population. However, not all the patients could bear the toxicity and cost of adjuvant therapies. The multimodal treatment strategies are still controversial now. For patients with a high risk of progression, additional adjuvant therapy should be considered to eliminate residual tumor cells and improve the survival outcomes. It is important to discover accessible indicators for high-risk patients and select appropriate therapeutic modalities.

Since the indices of blood cells are routinely tested at low cost, they may be suitable non-invasive biomarkers for clinicians. Various blood inflammatory biomarkers have been extensively studied in NSCLC. These factors correlated with inflammatory response and reflected the immune status of tumor microenvironment (TME). For example, neutrophils can secrete various inflammatory factors and up-regulate the tumorigenic and angiogenic factors, which creating an inflammatory environment favorable for tumor growth and metastases (13, 16). Platelets are involved in tumor development and progression through promoting angiogenesis (32). Monocytes were reported to promote tumor growth and angiogenesis, and inhibit the antitumor immune response (13, 33). By contrast, lymphocytes, particularly tumor-infiltrating lymphocytes (TILs), may induce cytotoxic cell death and inhibit tumor cell proliferation and migration (13). Thus, neutrophils, platelets and monocytes may have tumor-promoting properties and lymphocytes are essential for tumor defense and immune surveillance. The SII and SIRI are compound inflammatory biomarkers calculated by the counts of neutrophils, monocytes, platelets, and lymphocytes, which could better reflect the tumor immune microenvironment than any single index.

Meanwhile, blood inflammatory biomarkers could predict treatment outcomes and provide valuable information about the possibility of progression and survival time. In our study, we focused on the particular patient population of stage IIIA/N2 NSCLC treated with trimodality therapy and aimed to find potential blood inflammatory biomarkers as prognostic predictors and treatment indicators. We evaluated 34 patients and identified that low pretreatment SII (≤708.40), T2, and EGFR mutation-negative were indicator for longer DFS, and pretreatment SIRI (cutoff value: 0.82) was a prognostic factor for OS. We also analyzed the blood biomarkers of different times (after surgery, before chemotherapy, and before radiotherapy), but did not get any meaningful results. Overall, patients with high SII, high SIRI, T2 or EGFR mutation-negative have a high risk of poor prognosis and may need careful observation and forceful adjuvant treatment.

For oncogenic driver alteration NSCLC patients, the anti-tumor immune response in TME is always uninflamed. EGFR mutation-positive caused lack of TILs, impaired the antigen specific signal, made tumor cells unrecognizable to T cells, reduced programmed death receptor ligand-1 (PD-L1) expression, lower tumor mutation burden (TMB) (34–36). The immunosuppressive TME induced poor prognosis in EGFR mutation-positive NSCLC patients.

As for EGFR mutation-positive stage IIIA/N2 patients, adjuvant TKIs may be more beneficial than chemotherapy. In phase III ADJUVANT/CTONG1104 study (NCT01405079), gefitinib increased the DFS (28.7 months vs. 18.0 months, HR=0.60, 95% CI: 0.42-0.87, p=0.0054) compared with the chemotherapy group (vinorelbine plus cisplatin) in completely resected stage II-IIIA (N1-N2) EGFR mutation-positive NSCLC (37). The phase 3 ADAURA study (NCT02511106) assessed completely resected early-stage (stage II to IIIA) NSCLC patients with EGFR mutation-positive and reported that DFS was significantly longer in the osimertinib group than placebo group (not reached vs. 19.6 months; HR=0.17, 99.06% CI: 0.11-0.26, p<0.001) (38). There are also studies of adjuvant immunotherapy in the completely resected (stage II-IIIA) EGFR mutation-positive NSCLC patients (39). The best adjuvant treatment and the combination with radiotherapy are unknown in the light of current studies.

Our study has several limitations. First, it was a small, retrospective, single-center study with unavoidable bias. Second, blood inflammatory biomarkers could be influenced by various unidentified factors, such as active infection or concomitant use of nonsteroidal anti‐inflammatory drug or autoimmune disease. These factors could bring bias to results. Third, we used the R package SurvivalROC to get the cut-off values of blood inflammatory biomarkers in this study. The optimum method for proper cut-off values is uncertain. The cut-off values also vary in distinct patient population with specific treatment. Fourth, the anaplastic lymphoma kinase (ALK) rearrangement, and reactive oxygen species-1 (ROS-1) rearrangement were not validated because of the small study population. Future prospective studies with larger sample size and proper stratification are needed.

Despite the above limitations, our study was the first to investigate the relationship between blood inflammatory biomarkers and prognosis in stage IIIA/N2 NSCLC patients with trimodality therapy. Further prospective, large-scale studies are needed to further confirm the prognostic role of SII and SIRI. With the rapid development of immunotherapy, target therapy, and anti-vascular therapy, the best adjuvant treatment and corresponding cutoff values for distinct patients is unclear and requires more trials to investigate.

Furthermore, the importance of radiotherapy in enhancing anti-tumor immunity should be noticed in the design of prospective trials. To maximum the synergistic effect of radiotherapy, the radiation technique, target region and dose may be crucial in future prospective trials. T-lymphocytes are very radiosensitive. Larger radiation fields expose more lymphocytes to radiotherapy, which may in turn exhaust the T-cells. The degree of radiotherapy-induced lymphopenia is related to prognosis in NSCLC (40). However, the radiation region for stage III NSCLC patients usually includes draining lymph nodes, which affects the number and distribution of lymphocytes. Reduction in the volume of the radiation target (selective lymph nodal irradiation) and enhanced protection for normal lymph nodes with highly conformal techniques could reduce radiotherapy-induced lymphopenia. Maintaining the lymphocyte count may help to reduce the values of SII and SIRI and enhance anti-tumor immunity and eventually improve the survival of stage III NSCLC. The proper target region and the possibility of reduce the radiation dose to lymph nodes should be tested in future trials for a balance between lymphocytes reservation and local control of the tumor.

In conclusion, we identified that high pretreatment SII and SIRI were unfavorable prognostic factors in stage IIIA/N2 NSCLC patients treated with trimodality therapy. Pretreatment SII and SIRI may be potential indicators for further treatment options. Patients with high pretreatment SII, high pretreatment SIRI, T2, and EGFR mutation-positive may need more forceful adjuvant treatment. However, the proper adjuvant treatment is undecided. Further large-scale, prospective studies are needed to confirm our results, clarify the best cutoff values and most beneficial adjuvant treatment.

## Data Availability Statement

The raw data supporting the conclusions of this article will be made available by the authors, without undue reservation.

## Ethics Statement

The studies involving human participants were reviewed and approved by Ethics committee of the Affiliated Cancer Hospital of Zhengzhou University. Written informed consent for participation was not required for this study in accordance with the national legislation and the institutional requirements.

## Author Contributions

HY and LY designed this study and analyzed the data. HY and KW collected the data and wrote the manuscript. BL, YL, and SL assisted in collecting data and correcting the manuscript. All authors contributed to the article and approved the submitted version.

## Funding

This study was supported by Science and Technology Department, Henan Province (grant numbers: SB201901113 and 192102310048).

## Conflict of Interest

The authors declare that the research was conducted in the absence of any commercial or financial relationships that could be construed as a potential conflict of interest.

## Publisher’s Note

All claims expressed in this article are solely those of the authors and do not necessarily represent those of their affiliated organizations, or those of the publisher, the editors and the reviewers. Any product that may be evaluated in this article, or claim that may be made by its manufacturer, is not guaranteed or endorsed by the publisher.
